# Pitfalls of a Drug-Dispensing Audit Support System

**DOI:** 10.7759/cureus.89200

**Published:** 2025-08-01

**Authors:** Erika Hayashi, Takahiro Amemiya, Takashi Tomita

**Affiliations:** 1 Department of Pharmacy, Sanno Hospital, Minato-ku, JPN; 2 Department of Pharmaceutical Sciences, Teikyo Heisei University, Nakano-ku, JPN; 3 Department of Pharmacy, International University of Health and Welfare, Mita Hospital, Minato-ku, JPN; 4 Department of Pharmaceutical Sciences, School of Pharmacy at Narita, International University of Health and Welfare, Narita-shi, JPN

**Keywords:** c-correct ii®, dispensing audit support system, medical malpractice, pharmacy, weight errors

## Abstract

Dispensing audit support systems that authenticate pharmaceuticals via barcodes have been introduced into clinical practice to prevent dispensing errors, yet they occasionally generate false warnings, complicating clinical usage. This study sought to identify drugs prone to weight-error false warnings in the C-correct II^®^ dispensing audit support system and to clarify its operational problems. We investigated the drugs that caused false weight-error warnings in the system, confirming that such errors did occur. Furthermore, the drugs causing weight errors tended to be significantly heavier than those that did not. Accurately identifying and addressing such operational issues within dispensing audit support systems will minimize the risk of dispensing errors and ultimately enhance medical safety.

## Introduction

Dispensing errors can lead to serious incidents, making their prevention a critical task for pharmacists [1]. While attention has been given to drugs with similar names or varying dosages [2], reliance on human vigilance alone is insufficient to prevent errors. In fact, they cannot completely prevent dispensing errors [3]. To achieve safe medical care, there is an urgent need to develop a system that prevents dispensing errors. With the partial revision of the Implementation Guidelines for Barcode Labeling on Ethical Drugs issued by the Ministry of Health, Labour and Welfare in June 2012, barcodes (GS1 codes) are now also displayed on press-through package (PTP, a blister pack for oral drugs) sheets for oral drugs and tubes for external drugs [4]. In recent years, mechanization has progressed in dispensing operations, and dispensing audit support systems with barcode authentication have been developed [5]. C-correct II^®^ is a dispensing audit support system that uses the barcode attached to the PTP to accurately confirm the drug dispensed, converts the weight of the drug to its quantity, and checks for excess or deficient quantities [6]. Sanno Hospital introduced C-correct II^®^ in October 2024 to achieve safe auditing operations. Although the use of dispensing audit systems reduces the number of dispensing-related incidents, dispensing audit systems sometimes display incorrect warnings despite the correct drug and quantity [7]. There is a concern that reaudits due to false alerts will result in lost time due to increased audit procedures, which can be burdensome to auditors. Therefore, this study aimed to focus on the C-correct II^®^ audit data to identify drugs that are prone to false weight-related warnings and to understand the operational problems of the dispensing audit support systems. This study provides important suggestions for the operation of a safe auditing system that uses a dispensing audit support system.

## Technical report

Methodology

Drug-Dispensing Audit Support System

**Figure 1:**
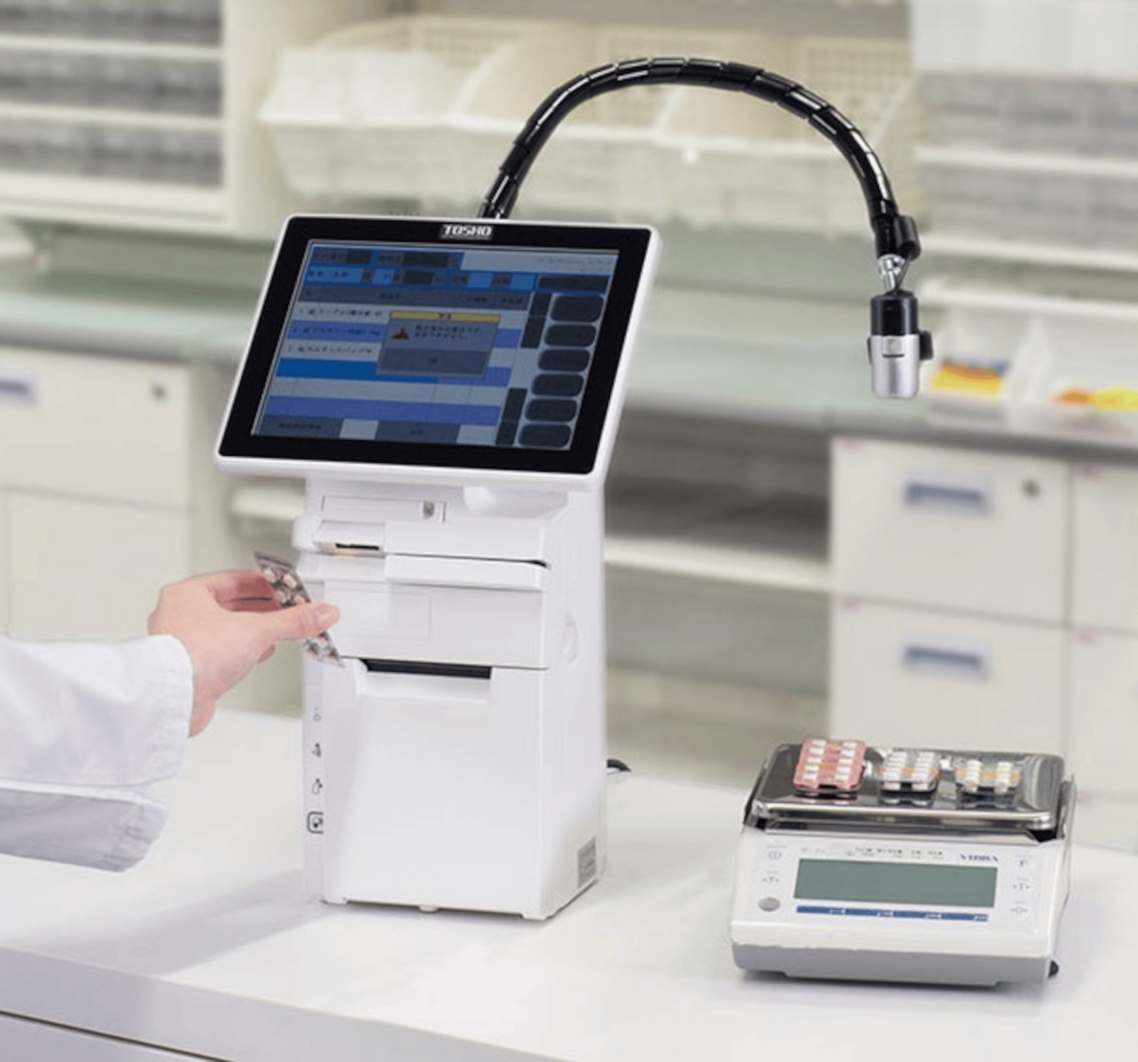
Overview of C-correct II®.

C-correct II^®^ (TOSHO Inc.) was used as the drug-dispensing audit support system (Figure 1) [6].

During normal operations, the barcode on the medicine bag is read using the C-correct II^®^’s scanner at the end of dispensing, and the prescription information is presented on the display. Next, the scanner reads the barcode of the drug and determines whether the drug type is correct. The drugs are then placed on a scale, and the quantity is checked based on the weight of the drugs. If the information on the prescription matches the quantity of the drug on the scale, it is automatically verified, and the system proceeds to verify the drug on the next prescription. The system is set up to alert the user with a screen display and an audible alert if the drug type or quantity is incorrect. When weight errors occur, a warning sound plays, and a message is displayed. In such cases, the drug-dispensing audit support system is run again from the beginning. If the number of doses is correct but the weight differs, it is determined to be a false weight-error warning. False weight-error warnings were extracted from the dispensing audit support system logs. The minimum unit of the scale is 0.01 g. In addition, the weight was converted to that of one tablet, which was registered as the standard weight. During the audit, the weight of the required number of tablets was calculated, and the allowable range of weight deviation before the alarm is activated was set to an error of ±3 tablets from the standard value.

Verification of Weight Errors in the Drug-Dispensing Audit Support System

We decided to focus on the average weight per drug dose when evaluating drug weight errors in this study, taking into account that the weight of each drug dose varies. Drugs that caused false weight-error warnings in the system in January 2025 were investigated and compared in the weight to drugs that did not cause weight errors.

Statistical Analysis

Significant differences in the drug weight comparisons were evaluated using the Student’s t-test (Excel®, Microsoft), with p-values <0.05 being considered a significant difference.

Ethical Considerations

The records of the drug-dispensing audit support system used in this study did not contain any personal information that posed any ethical issues. Thus, the need for informed consent was waived.

Results

Pitfall Assessment of Weight Errors in the Drug-Dispensing Audit Support System

**Figure 2:**
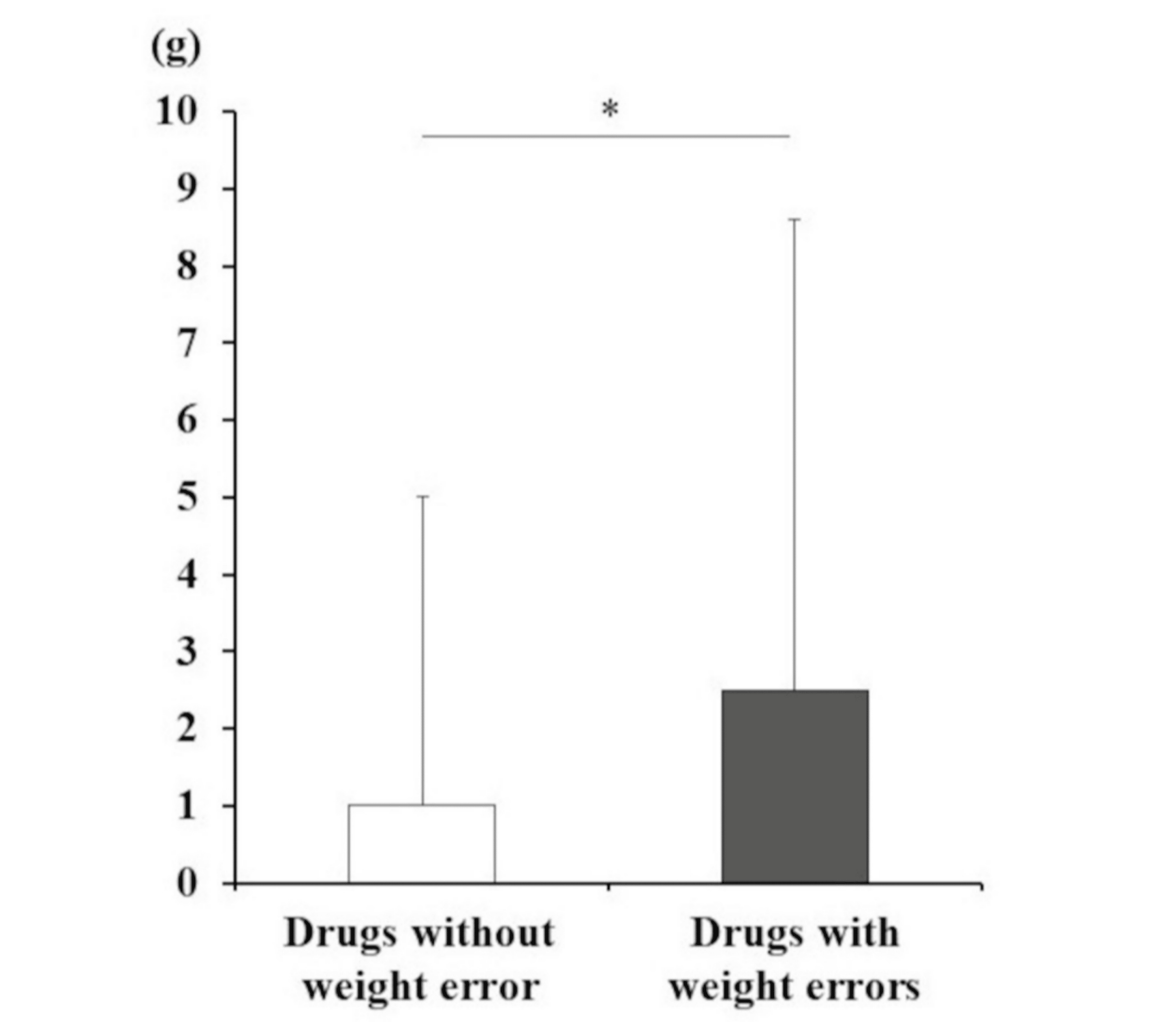
Weight comparison of drugs that caused weight errors and those that did not. Data are expressed as the mean ± SD. *: A p-value <0.05 represents a significant difference.

To develop a safe dispensing audit support system, it is important to focus on evaluating the system’s false warnings. In January 2025, 38 of 30,518 audited prescriptions triggered false weight-error warnings in the C-correct II^®^ system. The 38 items represent 38 individual drugs. Among the 38 items with weight errors, some medications had multiple weight errors, resulting in a false alarm rate of 0.56%. Furthermore, the weights of the drugs with weight errors (n = 38, 2.51 ± 6.10 g) were significantly higher (p = 0.03) than those without them (n = 651, 1.02 ± 4.00 g) (Figure 2).

## Discussion

In this study, we performed the first evaluation of drugs susceptible to weight errors during usage of the C-correct II^®^ dispensing audit support system. Dispensing audit support systems generally verify that drugs are selected as prescribed by matching the barcodes on the PTP and drug bag. When errors occur, the system alerts the user via a screen display and voice [1]. Implementing such dispensing audit support systems helps prevent drug errors [8]. In particular, the system eliminated several medication errors in our hospital, thereby demonstrating the usefulness of the dispensing audit support system. In addition to preventing such errors, implementing these systems may also improve operational efficiency. For example, their usage not only improves management quality by enabling retrospective verification of matching histories but can also be used to reduce the burden of ordering operations by controlling quantities [9]. In addition, if an audit camera is available when responding to patient inquiries, referring to the image record will reduce unnecessary re-issues, shorten response times, and reduce re-issue costs [10]. Furthermore, if a weight audit function is provided, the quantity of drugs, such as tablets and capsules, can be checked objectively according to the weight of the drug product, thereby preventing quantity errors [1]. Therefore, even in situations with limited staffing, such as at night or when on duty, the dispensing audit support system can help prevent errors in retrieving or counting medicines, contributing to the improved efficiency and accuracy of auditing operations [9].

Several challenges are associated with implementing the system. This study revealed that drugs with erroneous weight-error warnings in the dispensing audit support system had significantly higher weights than drugs without errors. The causes of these weight errors are unknown; however, it is assumed that heavier tablets come on larger sheets, so if fractions of sheets are required, cutting the PTP sheet will be more likely to introduce weight errors. Granules and powders were also among the drugs for which weight errors were observed. Many of our patients were chronically ill and were prescribed drugs for long periods. The possibility that small differences in the content of these drugs could lead to weight errors owing to long-term prescription is another possible consideration. High-frequency false alerts can cause alarm fatigue, reducing user awareness of the alerts and undermining trust in the system, which can lead to missed critical alerts and new medical errors [7]. Therefore, the settings at each facility must be adjusted to reduce the frequency of false positives.

One limitation of this study is that the analysis was performed for oral medications with high prescription volumes. In addition, C-correct II^®^ was used as the dispensing audit support system, and no other systems were evaluated as part of this study. Although this study was limited to one institution, the drug weight error was not limited to a single C-correct II^®^ but was found to be uniform across all C-correct II^®^ introduced. Additionally, this study did not examine false-negative weight errors. The 651 medications without weight errors referred to in this study were oral medications available at our hospital that have shown no weight errors as of January 2025. While most of these were dispensed over the study period, not all were. Finally, the sample size is not shown because this study was conducted at a single institution over a limited period.

## Conclusions

When drugs with weight errors were examined during usage of the C-correct II^®^ dispensing audit support system, it was found that drugs with weight errors were significantly heavier than those without weight errors. Accurately identifying and appropriately addressing operational issues that occur when using dispensing audit support systems can minimize the risk of dispensing errors and improve medical safety.
